# Robust Sampling of Defective Pathways in Multiple Myeloma

**DOI:** 10.3390/ijms20194681

**Published:** 2019-09-21

**Authors:** Juan Luis Fernández-Martínez, Enrique J. de Andrés-Galiana, Francisco Javier Fernández-Ovies, Ana Cernea, Andrzej Kloczkowski

**Affiliations:** 1 Group of Inverse Problems, Optimization and Machine Learning, Department of Mathematics, University of Oviedo, Oviedo 33007, Asturias, Spain; ej.deandres@gmail.com (E.J.d.A.-G.); javiovies@uniovi.es (F.J.F.-O.); cerneadoina@uniovi.es (A.C.); 2 Department of Computer Science, University of Oviedo, Oviedo 33007, Asturias, Spain; 3 Battelle Center for Mathematical Medicine, Nationwide Children’s Hospital, Columbus, OH 43205, USA; 4 Department of Pediatrics, The Ohio State University, Columbus, OH 43205, USA; 5 Future Value Creation Research Center, Graduate School of Informatics, Nagoya University, Nagoya 464-8601, Japan

**Keywords:** multiple myeloma, analysis of defective pathways, Fisher’s ratio sampler, holdout sampler, genetic signatures, chromosome 13 deletion prediction, genes, pathways

## Abstract

We present the analysis of defective pathways in multiple myeloma (MM) using two recently developed sampling algorithms of the biological pathways: The Fisher’s ratio sampler, and the holdout sampler. We performed the retrospective analyses of different gene expression datasets concerning different aspects of the disease, such as the existing difference between bone marrow stromal cells in MM and healthy controls (HC), the gene expression profiling of CD34+ cells in MM and HC, the difference between hyperdiploid and non-hyperdiploid myelomas, and the prediction of the chromosome 13 deletion, to provide a deeper insight into the molecular mechanisms involved in the disease. Our analysis has shown the importance of different altered pathways related to glycosylation, infectious disease, immune system response, different aspects of metabolism, DNA repair, protein recycling and regulation of the transcription of genes involved in the differentiation of myeloid cells. The main difference in genetic pathways between hyperdiploid and non-hyperdiploid myelomas are related to infectious disease, immune system response and protein recycling. Our work provides new insights on the genetic pathways involved in this complex disease and proposes novel targets for future therapies.

## 1. Introduction

Multiple myeloma (MM) is hematologic malignancy that results from clonal proliferation of plasma cells that help fighting infections by making antibodies that recognize and attack germs. Multiple myeloma causes cancer cells to accumulate in the bone marrow, where they crowd out healthy white and red blood cells, leading to fatigue and an inability to fight infections. Instead of producing necessary antibodies, the cancer cells produce abnormal proteins (monoclonal proteins) that can cause different complications, such as: Frequent infections, bone problems (pain, thinning and broken bones), reduced kidney function and anemia. Among the factors that may increase the risk of developing multiple myeloma are: Old age, male gender, African-American ethnicity, family history of MM, and history of a monoclonal gammopathy of undetermined significance (MGUS) in the blood. Nevertheless, the causes of MM are still not very well known.

Bergsagel and Kuehl, Corre et al. studied the genetic factors of MM. The current, widely accepted model of oncogenesis describes two different pathways: Hyperdiploidy and immunoglobulins heavy locus (IGH) translocations [1,2]. Hyperdiploidy is probably due to the missegregation of chromosomes during mitosis. Hyperdiploid myelomas are characterized by trisomy of certain odd numbered chromosomes (3, 5, 7, 9, 11, 15, 19). This is observed in up to 55% of the MM patients. Immunoglobulins recognize foreign antigens and initiate immune responses and the complement system. IGH translocations are observed in 40% to 50% of the MM patients. Non-hyperdiploid myeloma is characterized by translocations of the immunoglobulin heavy chain alleles at chromosome 14q32. Zhou et al. presented the molecular characterization of MM [3]. These genomic profiling analyses revealed that dysregulated expression of cyclin D might be a universal event in myelomagenesis. Cyclin D1 is a protein encoded by the CCND1 gene that is involved in regulating cell cycle progression. Despite of all the advances, MM still remains an incurable disease.

In this paper we present a retrospective analysis of different gene expression microarrays concerning different aspects of MM. In these types of problems concerning phenotype prediction, the number of samples is much higher than the number of probes, therefore the classification problem is highly underdetermined and thus, the uncertainty space generated by all the possible solutions (gene signatures) is huge. Having different sets of genes with similar predicted accuracies at disposal, the question of which set of genes gives the best explanation of the disease arises. This question needs to be answered in terms of pathways. For this purpose, we need to sample the uncertainty space of the phenotype prediction problem, exploring different datasets related to different aspects of the disease.

The defective pathways are sampled via two different algorithms: Fisher’s ratio [4] and holdout [5] samplers. These algorithms have been used to unravel the altered pathways involved in the metastasis in triple negative breast cancer outperforming Bayesian networks [6]—and recently in Parkinson disease [7] —to provide new insights about the defective pathways which are involved. The problem addressed in this paper does not consist in just solving the classification problem involved in phenotype prediction, but in finding the genetic pathways that are involved in the genesis and development of this disease, which is hampered by the high degree of under-determinacy of these kind of problems.

## 2. Results and Discussion

### 2.1. GSE85837

Bone marrow stromal cells (BMSCs) from patients with MM are functionally distinct from those with normal marrow. Bone marrow stromal cells (BMSCs) from MM patients enhance constitutive NF-κB activity in MM cells in conjunction with IL-8 [8]. The analysis of these differences could lead to improved understanding of MM and to improved treatment of MM patients.

Table S1 shows the list of most discriminatory genes of the MM vs. HC phenotype. The minimum-scale genetic signature contains only 6 genes (*EFNA3* and 5 *LOCX* genes) and provides a perfect classification (100% LOOCV predictive accuracy). Within this list, MTHFD2 and S100A3 were the two most under-expressed genes and *EGR1* and HSPA1A the most overexpressed. Apart from the *LOCX* genes, the most frequently sampled genes found by the Fisher’s ratio sampler were:

- EFNA3 which encodes a member of the ephrin (EPH) family, which is the largest subfamily of receptor protein-tyrosine kinases implicated in mediating developmental events (especially in the nervous system and in erythropoiesis).
- PCDH9 which encodes a member of the proto-cadherin family which is important in mediating cell adhesion in neural tissues.
- RESP2 is a protein coding gene involved in vesicle-mediated transport and clathrin-mediated endocytosis.

The holdout sampler found that the most important gene, according to posterior frequency, was *DPRX* (divergent-paired related homeobox) and *TTY19* (testis-specific transcript, Y-linked 19). In both cases, most of these genes belong to an extended list of most discriminatory genes shown in Table 1.

1. The pathway analysis performed with the most frequent sample found by the Fisher’s ratio (FR) and holdout samplers provide the results in Table 1.
2. The pathways related to overexpression are mainly associated with *O*-glycosylation of proteins; with synaptic cell-adhesion molecules (neurexins and neuroligins), and with interferon-alpha and beta signaling.
3. The pathways related to under-expression are mainly associated with different aspects of metabolism (bile acid, steroids, RNA and vitamin D), DNA repair, gene expression (transcription) of regulatory T lymphocytes (Tregs).
4. The FR sampler gives more importance to the interferon alpha and beta signaling, insulin secretion and vitamin D metabolism. Vitamin D deficiency is extremely common in multiple myeloma with 40% of patients having vitamin D levels in very deficient ranges [9,10].
5. The holdout sampler highlights the role of signaling by receptor tyrosine kinases and glycosylation, among others pathways. The importance of glycosylation in MM has been emphasized by Connolly et al. [11].

### 2.2. GSE24870

This dataset involves the analysis of the differences in the gene expression profiling of CD34+ cells in MM vs. HC. CD34 is a transmembrane phosphor-glycoprotein. The CD34+ population is responsible for most of the hematopoietic activity in bone marrow [12]. Szczepek et al. [13] analyzed CD34+ cells in the blood of patients with multiple myeloma, showing a relationship between CD34+ MM B-cells and malignant plasma cells. These authors concluded that CD34 may play an important role in the biology of myeloma by facilitating extravasation from blood, and thus the spread of myeloma through the skeletal system.

Bruns et al. [14] studied the multiple myeloma–related deregulation of bone marrow-derived CD34+ hematopoietic stem- and progenitor cells with respect to healthy individuals, showing deregulations of signaling cascades, most notably TGFβ signaling, and pathways involved in cytoskeletal organization, migration, adhesion, and cell-cycle regulation in MM patients.

Our analysis found that *SIAH1* is the most discriminatory gene, providing 100% LOOCV predictive accuracy. This gene has a Fisher ratio of 9.6, and encodes a protein (E3 ligase) involved in the proteasome-mediated degradation of specific proteins, the regulation of the cellular response to hypoxia and in the induction of apoptosis. Then, *DUSP10* and *ZNF675* have Fisher’s ratios of 9.1 and 7.3, respectively. *DUSP10* (dual specificity phosphatase 10) is a protein coding gene involved in cytokine signaling in the immune system and RET signaling (tyrosine kinase receptors). *ZNF675* (zinc finger protein 675) is a protein coding gene involved in ubiquitin protein ligase binding (Table S2).

1. The pathway analysis performed with the most frequently sampled found by the FR and holdout samplers provided in Table 2, below.
2. The pathways related to overexpressed genes are mainly associated to cellular responses to stress, protein arginine methylation, regulation of transcription of genes involved in the differentiation of myeloid cells by *RUNX1*, WNT signaling (signal transduction pathway) and metabolism of proteins.
3. The pathways related to under-expressed genes are mainly associated to transcriptional activity of *SMAD2/SMAD3*: *SMAD4*, and different mechanisms involved in the immune system response.
4. The Fisher sampler highlights the role of SUMOylation of proteins in gene expression regulation and transcriptional regulation by *RUNX1* and small RNAs.
5. The holdout sampler highlights the role of infectious disease (influenza and HIV infections), metabolism of RNA and cellular responses to stress.

The results shown here (GSE85837 and GSE24870) are different from those presented by Liu et al. [15] via Monte Carlo sampling and random forest, who identified IL-8 and EIF2 signaling as the main mechanisms involved in the inhibition of matrix metalloproteases. Although these results are partially different, since they refer to different aspects of the MM disease, they are important as they provide an insight on the molecular mechanisms involved at different levels and on possible causes.

### 2.3. GSE6477

For this dataset we have analyzed the genes/pathways that differentiate hyperdiploid vs. non-hyperdiploid myelomas, which are two different models of oncogenesis in MM. We have also analyzed the effect of chromosome 13 deletion. We achieved 91% LOOCV accuracy using the most 30 discriminatory genes shown in Table S3. The two most discriminatory genes found were *NCAM1* and *MIR5193* with fairly low Fisher’s ratios (around 0.65). This fact implies that this differentiation using gene expression is subtle. *NCAM1* encodes a cell adhesion protein which is a member of the immunoglobulin superfamily which is involved in the development of the nervous system, and in the expansion of T-cells and dendritic cells which play an important role in immune surveillance. *MIR5193* is involved in post-transcriptional regulation of gene expression affecting the stability and translation of mRNAs. Table 3 shows the main pathways involved, sampled via FR and holdout samplers. Some of these pathways have already appeared in Table 1 and Table 2. The main pathways involve infectious disease, immune system response and protein recycling.

Finally, the chromosome 13 deletion was also predicted with 91% LOOCV accuracy, with RBM26, ARGLU1 and ZC3H13 being the most discriminatory genes, and with Fisher’s ratios higher than 1.1 (Table S4). These genes are related to RNA binding, estrogen-dependent expressions of *ESR1* and MRNA splicing and RNA processing through methylation.

The pathways analysis provided the following results (Table 4):

1. The pathways associated to the overexpressed genes and the Fisher’s ratio sampler are mainly related to cell cycle, and protein degradation.
2. The pathways associated to the under-expressed genes and the holdout sampler are related to viral infections and immune system response.

## 3. Material and Methods

In this paper we performed the retrospective analysis of different datasets concerning different aspects of MM:

1. GSE85837 dataset collected at the Seoul National University College of Medicine to study the gene expression of bone marrow stromal cells from myeloma patients (https://www.ncbi.nlm.nih.gov/geo/query/acc.cgi?acc=GSE85837). This dataset contains 28 samples, 15 patients with plasma cell neoplasm and 13 controls, 9 of them are B-cell lymphoma patients with no evidence of bone marrow involvement and 4 patients with mild-to-moderate cytopenia without evidence of hematologic malignancies. RNA was extracted from cultured bone marrow stromal cells. The experiment used the Illumina HumanHT-12 V4.0 Gene Expression BeadChip and the aim was to investigate expression profiles of bone marrow stromal cells.
2. GSE24870 dataset [14] to study gene expression profiling of CD34+ subsets in multiple myeloma and healthy individuals. This dataset contains 43 samples genotyped on the Affymetrix Human Genome U133A 2.0 microarray: 20 healthy donors and 23 multiple myeloma patients. The cell types were: Hematopoietic stem cells, common myeloid progenitors, granulocyte/monocyte progenitors and megakaryocyte/erythroid progenitors. In this dataset 22,277 genetic probes were monitored.
3. GSE6477 dataset [15,16]. In this dataset the gene expression profile of hyperdiploid MM is compared to that of non-hyperdiploid myeloma to identify differentially expressed genes. Also, information concerning the deletion of chromosome 13 are given. This dataset contains 162 samples and 22,283 probes genotyped on the Affymetrix Human Genome U133A Array.

To understand the altered pathways in a disease we solved a phenotype prediction, which consisted of identifying the sets of gene signatures **g** that optimally separate the classes {*HC*(*Healthy controls*),*MM*(*Multiple Myeloma*)} in which the phenotype was divided, through the design of an operator in the form:

(1) $$L^{*}\left(g\right):g\in {\mathbb{R}}^{s}\to C=\left\{\mathrm{HC},\;\mathrm{MM}\right\}$$

In this case, we used the gene expressions in the transcriptome measured in a given set of genetic probes that depended on the platform that was used. The training data set was composed of an expression matrix **E** of different samples (MM patients and healthy controls). The rows in this matrix were the different samples that were monitored in the transcriptome and the columns are the set of genetic probes that were measured for each sample. Accordingly, we also had the observed class array $\left(c^{\mathrm{obs}}\right)$ at disposal that contains the classes of the set of samples annotated by medical experts.

Finding the discriminatory genetic signatures involved solving the optimization of the cost function that represents the prediction error, that is, the number of uncorrected samples predicted by the classifier $L^{*}$. The accuracy of the classifier is Acc(g)=100−minO(g). In this paper we use the leave-one-out cross-validation (LOOCV) and the 75/25 holdout accuracies.

(2) $$O\left(g\right)=\|L^{*}\left(g\right)-c^{\mathrm{obs}}{\|}_{1}$$

These kind of prediction problems are highly underdetermined since the number of monitored genetic probes is always much larger than the number of disease samples, and consequently, the associated uncertainty space of these problems is huge. They are composed by the sets of high predictive genetic networks with similar predictive accuracy:

(3) $$M_{tol}=\left\{g:\;O\left(g\right)\langle E_{tol}\to Acc\left(g\right)\rangle 100-E_{tol}\right\}$$

The high degree of under-determinacy of the learning problem (2) made the characterization of the involved biological pathways very ambiguous [17]. $M_{tol}$ contains the sets of genetic signatures that predict the phenotype with a predictive accuracy higher than $Acc_{min}=100-E_{tol}$ and accounts for the uncertainty in the phenotype prediction. Besides, the existing noise in data (expression matrix **E**) and in the class assignment $\left(c^{\mathrm{obs}}\right)$) provokes that the genetic signature with the highest predictive accuracy cannot explain the origin of the disease [18]. Therefore, the analysis of the set of high discriminatory genetic networks in $M_{tol}$ by means of sampling techniques should serve to unravel the mechanistic pathways that explain the disease development. This knowledge, if correct, should help to find new therapeutic targets and to repositioning of certain existing drugs.

The Fisher’s ratio sampler [4] enabled the sampling of the defective pathways, considering the discriminatory power of individual genes as measured by the Fisher’s ratio. For that purpose, the set of differentially expressed genes were first selected, and different networks were sampled with a prior probability of each gene proportional to its Fisher’s ratio. The predictive accuracy estimation of the networks was based on leave-one-out-cross-validation (LOOCV) using a nearest neighbor classifier [19]. In this sampler the complexity of the genetic network was established randomly taking into account the length (number of genes) of the small-scale genetic signature with the highest LOOCV accuracy. Finally, the posterior sampling frequencies of the main prognostic genes involved in these networks and the biological pathways were established using the set of genes with the highest sampling frequencies via Reactome.

The holdout sampler [20] generated different random 75/25 data bags (or holdouts), where 75% of the data in each bag was used for learning and 25% for blind validation. For each of these bags the small-scale genetic signatures were determined [17,19]. The posterior analysis was performed taking into account all the small-scale genetic signatures with high predictive validation accuracy, and served to establish the defective genetic pathways. The holdout sampler has also been successfully applied to perform the uncertainty analysis in different fields of science and technology [21,22].

Both samplers outperformed the Bayesian networks [6] in the analysis of the pathways involved in the metastasis of triple negative breast cancer. Besides, the probabilistic factorization of the uncertainty space of a phenotype prediction problem via Bayesian networks is not unique and establishing mechanistic conclusions based on the optimum Bayesian Network (BN) is similar compared to the use of the small-scale genetic signature. This approach might lead to partial and/or wrong conclusions.

## 4. Conclusions

Multiple myeloma is a very complex disease, and the underlying causes of MM are currently unknown. We presented a retrospective analysis of three different datasets concerning different aspects of this disease. The genetic pathways involved in the development of MM are very diverse and seem to be dependent on the aspect of the disease that is addressed, and on the bioinformatics methodology that it is used to perform the analysis. Nevertheless, our analysis has shown a consensus on some of the genetic pathways involved. The analysis in BMSCs cells highlighted pathways related to interferon alpha/beta signaling, signaling by receptor tyrosine kinases, vitamin D metabolism and glycosylation. The analysis in CD34+ cells highlighted mechanisms related to SUMOylation of chromatin organization proteins, infectious disease, transcriptional activity of SMAD2/SMAD3 and cellular responses to stress. Our analysis has also shown the importance of uncharacterized LOCXX genes which are very important to properly understand the MM progression. Finally, the differentiation of hyperdiploid MM and the prediction of the chromosome 13 deletion were achieved with very high accuracy, enabling an understanding of the disease progression. The analysis has shown a significant pathway diversity. While the under-expressed genes are related to immune response and viral infections; the overexpressed genes are mostly related to DNA repair, cell cycle, cellular response to stress, differentiation of myeloid cells, epigenetic modifications and metabolism of proteins and protein recycling. Among all these pathways, the genes involved in the differentiation of myeloid cells by RUNX1 transcription regulation seem to be an important target for the establishment of new therapies. We hope that the results of this research will provide deeper insights into the mechanisms involved in this complex disease.

## Figures and Tables

**Table 1:** GSE85837. Pathway analysis via Fisher’s ratio and holdout samplers.

Overexpressed Genes	Under-Expressed Genes	Fisher Sampler	Holdout Sampler
Defective B3GALTL	Bile acid and bile salt metabolism	Interferon alpha/beta signaling	Signaling by receptor tyrosine kinases
o-glycosylation of TSR domain-containing proteins	Formation of incision complex in GG-NER	Acetylcholine regulates insulin secretion	RAB GEFs exchange GTP for GDP on RABs
Neurexins and neuroligins	Metabolism of steroids	G alpha (q) signaling events	Opsins
Diseases associated with o-glycosylation of proteins	Global genome nucleotide excision repair (GG-NER)	Heme biosynthesis	Defective B3GALTL
Interferon alpha/beta signaling	SLBP independent processing of histone pre-mRNAs	Vitamin D (calciferol) metabolism	ABC transporters in lipid homeostasis
Protein-protein interactions at synapses	RUNX1 and FOXP3 control the development of regulatory T lymphocytes (Tregs)		COPII-mediated vesicle transport
Acetylcholine regulates insulin secretion	Heme biosynthesis		O-glycosylation of TSR domain-containing proteins
Pyrimidine catabolism	Vitamin D (calciferol) metabolism		ER to Golgi Anterograde Transport
Antimicrobial peptides	SLBP dependent processing of replication-dependent histone pre-mRNAs		Intrinsic pathway for apoptosis

**Table 2:** GSE24870. Pathway analysis via the Fisher’s ratio and Holdout samplers.

Overexpressed Genes	Under-Expressed Genes	Fisher Sampler	Holdout Sampler
Cellular responses to stress	Transcriptional activity of SMAD2/SMAD3: SMAD4	SUMOylation of chromatin organization proteins	Infectious disease
RMTs methylate histone arginines	Interleukin-4 and 13 signaling	RMTs methylate histone arginines	Processing of capped intron-containing pre-mRNA
RUNX1 regulates transcription of genes involved in the differentiation of HSCs	Downregulation of SMAD2/3:SMAD4 transcriptional activity	Transcriptional regulation by RUNX1	mRNA splicing - major pathway
Transcriptional regulation by RUNX1	Transcriptional regulation by E2F6	Transcriptional regulation by small RNAs	mRNA splicing
DNA damage/telomere stress induced senescence	Late phase of HIV lifecycle	RNA polymerase I promoter clearance	Influenza infection
Signaling by WNT	Toll-like receptor 3 (TLR3) cascade	HDMs demethylate histones	Cellular responses to stress
RUNX1 regulates genes involved in megakaryocyte differentiation and platelet function	MyD88-independent TLR4 cascade	Nonhomologous end-joining (NHEJ)	HIV infection
Nonhomologous end-joining (NHEJ)	TRIF(TICAM1)-mediated TLR4 signaling	SUMOylation of chromatin organization proteins	Influenza lifecycle
SUMOylation of chromatin organization proteins	Circadian clock	RMTs methylate histone arginines	Translation

**Table 3:** GSE6477. Hyperdiploid differentiation. Pathway analysis via the Fisher’s ratio and holdout samplers.

Overexpressed Genes	Under-Expressed Genes	Fisher Sampler	Holdout Sampler
Intra-Golgi and retrograde Golgi-to-ER traffic	Infectious disease	Asparagine N-linked glycosylation	Infectious disease
Asparagine N-linked glycosylation	L13a-mediated translational silencing of ceruloplasmin expression	ER to Golgi anterograde transport	Nonsense-mediated decay (NMD)
ER to Golgi anterograde transport	GTP hydrolysis and joining of the 60S ribosomal subunit	Interleukin-3, 5 and GM-CSF signaling	Nonsense mediated decay (NMD) enhanced by the exon junction complex (EJC)
XBP1(S) activates chaperone genes	Nonsense mediated decay (NMD) independent of the exon junction complex (EJC)	Transport to the Golgi and subsequent modification	SRP-dependent cotranslational protein targeting to membrane
mRNA 3’-end processing	Eukaryotic translation initiation	Interleukin receptor SHC signaling	Nonsense mediated decay (NMD) independent of the exon junction complex (EJC)
IRE1alpha activates chaperones	Cap-dependent translation initiation	COPII-mediated vesicle transport	Regulation of expression of SLITs and ROBOs
Golgi-to-ER retrograde transport	Formation of a pool of free 40S subunits	CLEC7A (Dectin-1) induces NFAT activation	Eukaryotic translation initiation
Unfolded protein response (UPR)	SRP-dependent cotranslational protein targeting to membrane	AKT phosphorylates targets in the cytosol	Cap-dependent translation initiation
COPI-mediated anterograde transport	Peptide chain elongation	TP53 regulates transcription of genes involved in G1 cell cycle arrest	L13a-mediated translational silencing of geruloplasmin expression

**Table 4:** GSE6477. Chromosome 13 deletion prediction. Pathway analysis via the Fisher’s ratio and holdout samplers.

Overexpressed Genes	Under-Expressed Genes	Fisher Sampler	Holdout Sampler
The role of GTSE1 in G2/M progression after G2 checkpoint	Nuclear pore complex (NPC) disassembly	Cdc20:Phospho-APC/C mediated degradation of cyclin A	HIV infection
Assembly of the pre-replicative complex	ISG15 antiviral mechanism	APC:Cdc20 mediated degradation of cell cycle proteins prior to satisfaction of the cell cycle checkpoint	Glucuronidation
Orc1 removal from chromatin	Antiviral mechanism by IFN-stimulated genes	APC/C:Cdc20 mediated degradation of mitotic proteins	G2/M transition
Cdc20:Phospho-APC/C mediated degradation of cyclin A	NS1 mediated effects on host pathways	Activation of APC/C and APC/C:Cdc20 mediated degradation of mitotic proteins	Mitotic G2-G2/M phases
APC:Cdc20 mediated degradation of cell cycle proteins prior to satisfaction of the cell cycle checkpoint	Host interactions with influenza factors	Regulation of APC/C activators between G1/S and early anaphase	HIV lifecycle
APC/C:Cdc20 mediated degradation of mitotic proteins	Interferon signaling	APC/C-mediated degradation of cell cycle proteins	Infectious disease
Activation of APC/C and APC/C:Cdc20 mediated degradation of mitotic proteins	TNF signaling	Regulation of mitotic cell cycle	Cdc20:Phospho-APC/C mediated degradation of cyclin A
Mitotic G1-G1/S phases	Death receptor signaling	Regulation of mRNA stability by proteins that bind AU-rich elements	Host interactions of HIV factors
The role of GTSE1 in G2/M progression after G2 checkpoint	mRNA decay by 3’ to 5’ exoribonuclease	Separation of sister chromatids	APC:Cdc20 mediated degradation of cell cycle proteins prior to satisfaction of the cell cycle checkpoint

## Supplementary Materials

Supplementary materials can be found at https://www.mdpi.com/1422-0067/20/19/4681/s1.
