# Supplementary material for: Robust Sampling of Defective Pathways in Multiple Myeloma
Source: Int J Mol Sci. 2019 Sep 21;20(19):4681. doi: 10.3390/ijms20194681 (PMC6801400; doi:10.3390/ijms20194681)
Supplement: Supplementary file 1 [file ijms-20-04681-s001.pdf]

Robust Sampling of Defective Pathways in Multiple Myeloma

Juan Luis Fernández-Martínez, Enrique J. de Andrés-Galiana, Francisco Javier Fernández-Ovies , Ana Cernea, Andrzej Kloczkowski

Table S1. GSE85837. List of most discriminatory genes in Multiple Myeloma. HC stands for healthy controls and MM for Multiple Myeloma patients. In bold face the LOCXX genes are highlighted.

Gene	Mean-HC	Std-HC	Mean-MM	Std-HC	FR	Accuracy
EFNA3	7.23	0.13	7.43	0.12	2.27	71.4
LOC654078	7.26	0.15	7.5	0.12	2.06	82.1
LOC732313	7.38	0.11	7.17	0.14	1.96	85.7
LOC154822	7.35	0.13	7.09	0.12	1.94	92.9
LOC340096	7.29	0.1	7.45	0.12	1.9	92.9
LOC100101266	7.37	0.12	7.57	0.09	1.86	100
PCDH9	7.48	0.07	7.34	0.12	1.77	100
REPS2	6.93	0.11	6.81	0.09	1.67	100
LOC651896	7.16	0.1	7.33	0.14	1.64	100
RCVRN	7.32	0.16	7.46	0.09	1.6	100
LOC100130549	7.31	0.13	7.46	0.08	1.59	100
LOC100134744	7.1	0.13	6.92	0.15	1.56	100
LOC645405	7.41	0.14	7.19	0.18	1.53	100
B4GALNT2	6.62	0.12	6.78	0.15	1.53	100
FLJ37453	7.23	0.16	7.4	0.14	1.52	100
ZC3H14	6.52	0.1	6.71	0.16	1.48	100
DPRX	6.91	0.08	6.79	0.07	1.47	100
EXOG	7.44	0.08	7.28	0.13	1.47	100
LOC728846	7.23	0.11	7.08	0.16	1.47	100
LOC100129054	7.34	0.1	7.2	0.1	1.45	100
LOC643713	7.58	0.1	7.66	0.07	1.37	100
EIF4EBP3	6.83	0.11	6.98	0.15	1.35	100
RSAD2	7.38	0.1	7.55	0.1	1.34	100
LOC100129781	8.27	0.1	8.05	0.14	1.32	100
SEC15L2	7.5	0.15	7.32	0.1	1.32	100
RFX3	7.09	0.21	7.29	0.12	1.31	100
LOC653227	7.21	0.16	7.4	0.12	1.3	100
LOC650599	7.44	0.16	7.27	0.17	1.28	100
LOC100130451	7.47	0.13	7.32	0.11	1.27	100
LOC728393	7.34	0.1	7.43	0.14	1.26	100
MIR891A	6.96	0.14	6.78	0.15	1.26	100
OR52A1	6.49	0.08	6.62	0.12	1.25	100
ORM1	7.35	0.16	7.17	0.1	1.25	100
CST4	7.26	0.14	7.39	0.08	1.24	100
LOC100133840	8.52	0.37	8.25	0.23	1.24	100
LOC645139	7.28	0.14	7.4	0.15	1.24	100
LOC284232	7.41	0.16	7.28	0.15	1.23	100
PTH	7.37	0.12	7.21	0.19	1.23	100
GPR4	6.59	0.09	6.74	0.13	1.21	100

Table S2. GSE24870. List of most discriminatory genes in Multiple Myeloma. HC stands for healthy controls and MM for Multiple Myeloma patients.

Gene	Mean-HC	Std-HC	Mean-MM	Std-HC	FR	Accuracy
SIAH1	1492.76	157.47	732.54	190.41	9.54	100
DUSP10	1551.94	265.98	550.52	196.17	9.07	100
ZNF675	966.21	114.5	560.9	129.77	7.3	100
SKIL	1377.16	492.33	301.08	374.62	4.9	100
HIF1A	1734.75	342.97	907.48	188.92	4.88	100
BOLA2	3287.72	456.94	1950.35	433.02	4.72	100
EWSR1	340.9	70.27	541.11	98.19	4.24	100
RANBP9	2483.48	411.18	1557.04	322.21	4.2	100
SRSF10	2442.5	294.75	1564.54	254.76	4.17	100
PNRC2	4828.98	545.62	3282.73	546.81	4.12	100
SAP18	5575.27	693.24	3892.6	619.04	3.98	100
SP3	3654.02	358.57	2628.63	423.08	3.96	100
RLF	555.03	93.06	293.84	101.66	3.86	100
DAZAP2	7054.34	944.96	4838.82	976.8	3.8	100
EMP1	2161.81	653.22	870.19	440.99	3.72	100
YTHDF3	1020.97	168.51	661.45	141.92	3.7	100
KRR1	1720.96	206.06	1096.02	215.62	3.68	100
VCPKMT	640.05	142.3	328.6	111.56	3.64	100
BCLAF1	1706.19	280.71	997.64	199.14	3.62	100
CSRP1	557.23	167.37	1014.72	197.07	3.6	100
FKBP2	848.76	169.22	1572.74	405.16	3.55	100
ACAP2	1742.66	265.43	1032.09	233.19	3.5	100
RPL8	6703.09	974.89	10375.96	1736.2	3.48	100
BTG3	945.9	164.61	515.79	186.12	3.47	100
RNF138	3598.19	307.16	2375.59	585.09	3.47	100
NECAP1	289.12	49.65	164.72	38.53	3.46	100
ZNHIT1	357.15	115.73	734.59	154.49	3.44	100
RGS1	2616.15	657.61	1012.82	910.61	3.37	100
ALDOA	1471.41	393.3	2609	570.92	3.36	100
CD69	6679.83	1473.33	3158.37	1163.1	3.36	100
RGS1	1656.65	543.27	527.12	430.49	3.35	100
PSPH	1131.34	509.58	259.17	95.61	3.28	100
ARL4A	3766.28	698.13	2220.35	698.69	3.28	100
MARCH7	3771.23	568.36	2373.61	583.58	3.27	100
BCAP31	588.68	175.22	1210	365.28	3.25	100
ALDOA	1653.02	397.55	2900.39	653.88	3.25	100
SNRNP70	2140.91	225.99	3368.45	655.35	3.24	100
DUSP6	3173.01	929.38	1396.33	426.89	3.23	100
MED13L	2187.96	322.75	1378.38	235.8	3.21	100

Table S3. GSE6477. List of most discriminatory genes in the Hyper-diploid differentiation. HD stands for Hyperdiploid and NHD for Non-Hyperdiploid.

Gene	Mean-HD	Std-HD	Mean-NHD	Std-NHD	FR	Accuracy
NCAM1	1751.61	1169.25	890.58	876.06	0.64	67.74
MIR5193	809.44	378.63	503.5	367.85	0.63	70.32
DYNLT3	648.12	170.14	816.8	195.85	0.6	74.84
ANXA7	838.13	280.48	1091.48	279.86	0.55	82.58
RPN2	12529.96	4573.32	17045.27	5056.27	0.53	82.58
RBMS1	682.86	374.45	930.39	406.3	0.5	86.45
GTF2H1	675.62	190.22	536.04	132.33	0.49	85.81
TMED2	6556.89	1938.02	8494.5	2313.89	0.49	87.1
ANP32A	598.76	207.93	402.75	150.5	0.49	85.81
RBMS1	881.11	501.31	1455.24	716.63	0.49	88.39
PIK3R3	386.39	212.85	199.4	124.38	0.48	88.39
SLTM	995.04	310.36	728.98	243.03	0.48	90.32
TMX4	417.56	223.14	721.24	386.87	0.47	89.68
IVD	396.18	161.79	275.07	128.13	0.47	90.32
CHMP6	149.99	89.22	217.53	103.36	0.46	90.32
RBMS1	1075.15	464.03	1567.75	642.59	0.46	90.97
KCNN3	670.95	809.75	1265.95	898.83	0.46	90.97
CLTA	2241.79	727.21	1612.5	610.42	0.45	90.97
GYG1	2273.96	900.78	1579.71	676.35	0.45	89.68
IDH2	4933.21	2652.22	2801.42	1685.73	0.45	87.74
TBCE	457.33	122.66	570.83	141.69	0.44	89.03
SNORA11E	160.87	177.77	313.1	267.12	0.44	89.68
TMBIM6	4390.19	1397.85	6202.94	2070.17	0.44	92.26
NPM1	12693.69	5693.08	8904.49	3273.24	0.43	90.32
ACTR1A	529.6	189.74	681.85	167.87	0.43	90.32
MXI1	1715.98	573.71	2280.57	815.38	0.43	89.68
RAB5B	692.88	156.29	892.08	195.69	0.42	90.32
GCOM1	610.35	212.59	465.32	117.51	0.42	89.68
ASXL1	786.84	187.64	960.9	221.83	0.42	88.39
**CD63**	**8510.71**	**3333.8**	**12647.6**	**4976.83**	**0.42**	**90.97**
CSNK1A1	1294.35	399.71	1041.05	345.01	0.42	89.03
FXR1	1243.72	508.89	915.4	441.91	0.41	87.74
IDH2	855.2	272.45	600	208.22	0.41	87.1
LDLRAP1	555.8	291.32	849.05	376.97	0.41	89.03
COG2	864.35	208.97	1043.07	245.19	0.4	89.03
PAK1	147.96	87.25	96.02	78.15	0.4	89.03
NSL1	585.37	201.89	763.87	252.35	0.4	89.68
SEC31A	2726.55	1104.94	3360.84	1004.18	0.4	90.32
GTF3C1	260	174.76	394.1	208.92	0.4	90.97
YWHAZ	4381.4	1183.21	5665.59	1821.07	0.4	90.97
RPL13A	24183.75	13371.77	14671.14	7879.12	0.4	89.68
PPP6C	1692.43	474.29	1314.39	468.99	0.4	89.03
RPN2	14438.29	6054.34	19511.13	6307.89	0.39	89.68

Table S4. GSE6477. List of most discriminatory genes to predict the Chromosome-13 deletion. N stands for for normal and DEL for Ch-13 deleted.

Gene	Mean-N	Std-N	Mean-DEL	Std-DEL	FR	Accuracy
RBM26	1113.38	321.33	677.93	240.41	1.22	74.68
ARGLU1	1178.26	409.25	722.82	330.49	1.07	77.85
ZC3H13	464.93	128.74	314.06	93.97	1.07	79.75
UFM1	1509.17	428.15	1007.8	342.35	0.88	79.75
MYCBP2	1253.62	447.9	687.83	399.68	0.88	80.38
STK24	2271.41	547.91	1613.18	562.56	0.86	82.28
UCHL3	720.08	196.32	474.41	228.97	0.85	82.91
ZMYM2	674.39	209.59	457.52	172.79	0.83	82.28
CDC16	855.43	253.69	538.59	273.36	0.83	82.28
MYCBP2	1824.1	545.51	1151.14	656.8	0.82	83.54
GTF3A	3645.07	1016.91	2432.57	920.06	0.79	84.81
PHF11	842.41	270.26	583.16	253.22	0.78	82.91
ALG11 /// UTP14C	808.96	189.66	624.59	150.66	0.71	84.81
ANKRD10	747.47	227.57	478.48	175.61	0.71	84.81
CDC16	1173.68	326.46	876.13	334.85	0.66	82.91
FOXO1	1004.06	375.62	703.08	311.25	0.65	82.91
MED6	102.42	59.03	64.01	52.45	0.59	85.44
N4BP2L2	451.02	152.35	325.83	143.05	0.57	85.44
NEK3	318.63	157.44	197.42	128.5	0.57	84.81
NDUFA9	1170.88	279.47	1488.11	356.56	0.57	84.18
PDS5B	440.62	134.51	326.27	112.69	0.55	83.54
TGDS	707.29	198.2	529.65	184.17	0.55	84.81
USP12	302.94	94.61	217.27	79.15	0.55	84.81
C1orf112	64.93	59.3	102.57	76.92	0.52	84.81
SUPT20H	554.76	149.03	422.97	149.25	0.49	84.81
C6orf211	186.75	75.51	254.66	100.65	0.48	86.08
RB1	710.29	355.66	434.47	340.02	0.48	86.08
DICER1	539.02	208.06	412.82	161.52	0.48	87.34
GTF3C1	260.01	170.25	408.11	210.14	0.47	86.08
MPZL1	480.28	194.51	659.46	324.81	0.47	86.08
RBM25	882.72	321.9	654.1	281.93	0.47	86.71
NAA16	404.46	153.78	258.42	96.8	0.47	86.71
OGT	2998.6	1135.25	2011.19	1029.35	0.47	86.71
PCID2	748.44	153.85	606.26	172.55	0.47	86.71
TPP2	837.33	256.8	647.28	248.96	0.47	87.34
STK24	805.24	215.06	622	196.7	0.46	87.34
CDC16	1182.95	306.31	864.16	351.92	0.45	87.34
N4BP2L2	1054.55	591.22	517.78	408.26	0.45	87.34
AKAP11	870.41	288.01	644.58	259.24	0.45	86.71
OGT	3429.98	1228.66	2583.22	1266.24	0.44	87.34
RCBTB2	1007.42	505.58	592.01	364.79	0.44	86.71
CLN	296.03	90.19	239.54	103.92	0.44	87.34
OGT	5000.14	1722.38	3800.23	1415.94	0.44	86.71
